# Overcoming acquired resistance to cancer immune checkpoint therapy: potential strategies based on molecular mechanisms

**DOI:** 10.1186/s13578-023-01073-9

**Published:** 2023-06-30

**Authors:** Bin Wang, Yin Han, Yuyu Zhang, Qin Zhao, Huanhuan Wang, Jinlong Wei, Lingbin Meng, Ying Xin, Xin Jiang

**Affiliations:** 1 Department of Radiation Oncology, The First Hospital of Jilin University, 71 Xinmin Street, Changchun, 130021 China; 2 Medical Oncology, Cancer Center, West China Hospital, Sichuan University, Chengdu, 610041 China; 3 Cancer Prevention and Treatment Institute of Chengdu, Department of Pathology, Chengdu Fifth People’s Hospital (The Second Clinical Medical College, Affiliated Fifth People’s Hospital of Chengdu University of Traditional Chinese Medicine), Chengdu, 611137 China; 4 Jilin Provincial Key Laboratory of Radiation Oncology & Therapy, The First Hospital of Jilin University, Changchun, 130021 China; 5 NHC Key Laboratory of Radiobiology, School of Public Health, Jilin University, Changchun, 130021 China; 6 Department of Hematology and Medical Oncology, Moffitt Cancer Center, Tampa, FL 33612 USA; 7 Key Laboratory of Pathobiology, Ministry of Education, Jilin University, 126 Xinmin Street, Changchun, 130021 China

**Keywords:** Immune checkpoint inhibitors, Acquired resistance, Mechanisms, Tumor microenvironment, Combination therapy

## Abstract

Immune checkpoint inhibitors (ICIs) targeting CTLA-4 and PD-1/PD-L1 to boost tumor-specific T lymphocyte immunity have opened up new avenues for the treatment of various histological types of malignancies, with the possibility of durable responses and improved survival. However, the development of acquired resistance to ICI therapy over time after an initial response remains a major obstacle in cancer therapeutics. The potential mechanisms of acquired resistance to ICI therapy are still ambiguous. In this review, we focused on the current understanding of the mechanisms of acquired resistance to ICIs, including the lack of neoantigens and effective antigen presentation, mutations of IFN‐γ/JAK signaling, and activation of alternate inhibitory immune checkpoints, immunosuppressive tumor microenvironment, epigenetic modification, and dysbiosis of the gut microbiome. Further, based on these mechanisms, potential therapeutic strategies to reverse the resistance to ICIs, which could provide clinical benefits to cancer patients, are also briefly discussed.

## Introduction

Over the last decade, the emergence of anticancer immunotherapies, especially immune checkpoint inhibitors (ICIs) targeting cytotoxic T lymphocyte antigen 4 (CTLA-4), programmed death 1 (PD-1), or PD-ligand 1(PD-L1) to boost tumor-specific T lymphocyte immunity, has opened a brand new chapter for treatment of multiple histological types of malignancies with durable responses and unsurpassed clinical efficacy [1]. Compared to chemotherapeutic agents or targeted therapies, ICIs are characterized by a persistent response that can be translated into long-term survival benefits. A pooled analysis from clinical trials revealed that advanced melanoma patients treated with ICIs, such as ipilimumab and pembrolizumab, exhibited durable clinical responses, as evidenced by a three-year survival rate of about 70% and an overall survival (OS) exceeding 10 years in over 21% patients [2, 3].

Despite the unprecedented durable responses and survival benefits that have been observed, the majority of patients are less sensitive to ICI monotherapy, demonstrating primary resistance. The objective response rates of ICI monotherapy seldom exceed 40% for most tumor types (and are generally much beneath this figure), with a large proportion of patients having a partial response [4–6]. And more worryingly, an encouraging initial decrease in overall tumor burden observed in a number of patients can be offset by the evolution of acquired resistance to ICI therapy, which eventually leads to radiological and/or clinical disease progression [7, 8]. A study showed that approximately 25% of melanoma patients developed acquired resistance following an initial response to pembrolizumab, with disease progression evident during the median follow-up of 21 months [9]. Another study also suggested that after a five-year follow-up, 39% of patients with melanoma who initially responded to nivolumab had disease progression [10]. Similarly, more than 30% non-small cell lung cancer (NSCLC) patients were found to have relapsed after an objective initial response to nivolumab at the 2-year follow-up [11]. Accordingly, the pooled analysis of four clinical trials of nivolumab in NSCLC patients revealed that up to 65% of responders had progressed at 4 years follow-up [12]. Several studies attempted to infer the incidence of acquired resistance by analyzing durable response data, ranging from 11 to 77% across different tumor types [13]. However, up to now, no uniform definition has been established for acquired resistance to ICI therapy. Although the immunotherapy resistance taskforce of the Society for Immunotherapy of Cancer (SITC) considered acquired resistance as the occurrence of disease progression in the setting of ongoing treatment in the patient who had previously achieved a documented, confirmed objective response or stable disease lasting for more than 6 months following antineoplastic therapy, the response evaluation criteria remain divided [8] As in contrast to primary resistance various mechanisms of which have been identified during the past decade, until recently, mechanisms of acquired resistance remain ambiguous, thereby few reliable predictive biomarkers and effective treatment options can be used in such patients [14]. In this review, we outlined the present understanding of mechanisms underlying acquired resistance to ICI therapy and briefly explored potential strategies to counter the resistance and improve overall outcomes from the perspective of resistant mechanisms.

## The cancer immunity cycle and acquired resistance to immune checkpoint inhibitors

**Fig. 1:**
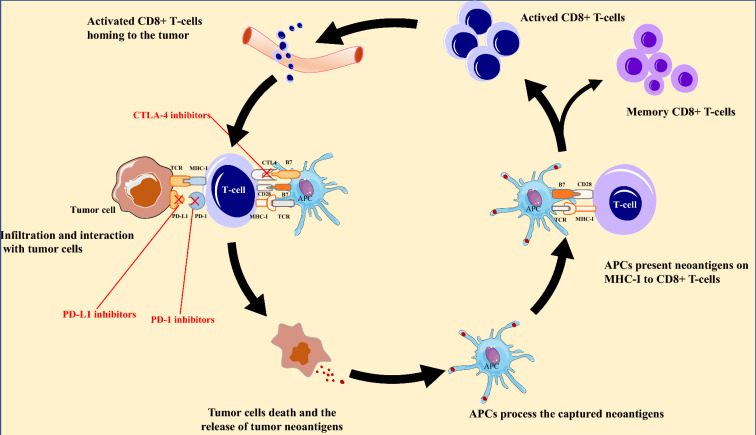
Anti-tumor immune response and ICIs. The induction of the effective anti-tumor immune response requires multiple steps: (I) The tumor-associated neoantigens are released and subsequently captured by APCs; (II) APCs present the captured antigens on MHCI molecules to T-cells, resulting in the proliferation and activation of T-cells, (III) homing and infiltration of activated T-cells. (IV) recognition of peptide MHC-I complexes and release of perforin and lytic granules to mediate tumor cell killing. Abnormalities in each of these steps during or after treatment can develop acquired resistance to ICI therapy

The process of anti-tumor immunity is a complex sequence of multiple steps, which can be defined as the “Cancer-Immunity Cycle” (Fig. 1). First, the released tumor-associated neoantigens are captured by the antigen-presenting cells (APCs), subsequently migrating to secondary lymphoid organs as well as to tumor-related tertiary lymphoid structures [15]. Second, the APCs process the captured neoantigens into immunogenic polypeptides and present them to CD8^+^ T cells via the binding of the antigen peptide-major histocompatibility complex class I (MHC-I) molecule complex on the APC surface and the T cell receptor (TCR) on the surface of native CD8^+^ T cells, which leads to the activation and proliferation of CD8^+^ T cells [16]. The engagement of costimulatory molecules, CD28 and B7, is a necessary condition for the complete activation of naive T cells, and is strictly regulated by inhibitory immune checkpoints like CTLA-4 or PD-1 and their ligands. Third, the activated CD8^+^ T‐cells home-in to the tumor by extravasating via the endothelium and infiltrating through the surrounding stromal tissue [17]. Lastly, the TCRs on the surface of infiltrating CD8^+^ T‐cells need to establish contact with the peptide MHC-I complexes on the surface of APCs to release perforin and lytic granules in the immune synapse, thereby mediating tumor destruction [18]. After tumor cells are eradicated, memory T cells are formed which keep quiescent until a re-exposure to the neoantigens [19]. Under natural conditions, immune checkpoints play a negative feedback role in regulating immune responses after T-cell activation. CTLA-4 on T cells binds to B7 ligands on APCs with much higher affinity and avidity than CD28, which competitively interferes with CD28-B7 interactions, thereby preventing costimulation at the T-cell-APC interface and inhibiting activation of T cell responses [20]. Interaction of PD-1 with its ligands (PD-L1/PD-L2) can inhibit the effector stage of T-cell activation, thus suppressing the immune response [6]. The continuous interaction between inhibitory and stimulatory signals promotes adaptive responses against tumor-associated neoantigens while avoiding autoimmunity [21]. It has been confirmed that various tumors escape T-cell killing by hijacking this mechanism, and antibodies directly targeting CTLA-4, PD-1, and PD-L1 have exhibited significant anti-tumor responses. Thus far, eight ICIs inhibitors for CTLA-4 (ipilimumab), PD-1 (nivolumab, pembrolizumab, cemiplimab, and dostarlimab), and PD-L1 (durvalumab, avelumab, and atezolizumab) have been approved by the U.S. Food and Drug Administration (FDA) for the management of over 20 cancer types, the clinical indications of which are given in Table 1 [22]. Relatlimab targeting the lymphocyte-activation gene 3 (LAG-3) received its first approval by the FDA for the treatment of metastatic or unresectable melanoma in March 2022 [23]. Agents inhibiting other immune checkpoints like are still in clinical testing [24]. Abnormalities in any step of the “Cancer-Immunity Cycle” during or after treatment can result in ICI acquired resistance.

**Table 1:** Summary of FDA-approved immune checkpoint inhibitors

Agent	Manufacturer	Target	Molecular type	FDA-Approved Application	Trail(s) based
Pembrolizumab (Keytruda)	Merck & Co	PD-1	An IgG4 kappa immunoglobulin	Melanoma	KEYNOTE-002 (NCT01704287) KEYNOTE-006 (NCT01866319) KEYNOTE‑054 (NCT02362594)
				NSCLC	KEYNOTE‑042 (NCT02220894) KEYNOTE-189 (NCT02578680) KEYNOTE-407 (NCT03875092) KEYNOTE-021 (NCT02039674) KEYNOTE-010 (NCT01905657)
				HNSCC	KEYNOTE-048 (NCT02358031) KEYNOTE-012 (NCT01848834)
				cHL	KEYNOTE-204 (NCT02684292) KEYNOTE-087 (NCT02453594)
				PMBCL	KEYNOTE-170 (NCT02576990)
				Urothelial carcinoma	KEYNOTE-052 (NCT02335424) KEYNOTE-045 (NCT02256436)
				Colorectal cancer	KEYNOTE-177 (NCT02563002)
				Gastric cancer	KEYNOTE-811 (NCT03615326)
				Esophageal cancer	KEYNOTE-590 (NCT03881111) KEYNOTE-181 (NCT03933449)
				Cervical cancer	KEYNOTE-826 (NCT03635567) KEYNOTE 158 (NCT02628067)
				HCC	KEYNOTE 224 (NCT02702414)
				MCC	KEYNOTE-017 (NCT02267603)
				RCC	KEYNOTE-426 (NCT02853331) KEYNOTE-581 (NCT02811861) KEYNOTE‑564 (NCT03142334)
				Endometrial carcinoma	KEYNOTE 158 (NCT02628067) KEYNOTE-775 (NCT03517449)
				TMB-high cancer	KEYNOTE 158 (NCT02628067)
				cSCC	KEYNOTE-629 (NCT03284424)
				TNBC	KEYNOTE-522 (NCT03036488) KEYNOTE-355 (NCT02819518)
Nivolumab (Opdivo)	Bristol-Myers Squibb	PD-1	A human IgG4 monoclonal antibody	Melanoma	CHECKMATE-037 (NCT01721746) CHECKMATE-066 (NCT01721772) CHECKMATE-067 (NCT01844505) CHECKMATE-238 (NCT02388906)
				NSCLC	CHECKMATE-227 (NCT02477826) CHECKMATE-816 (NCT02998528) CHECKMATE-9LA (NCT03215706) CHECKMATE-057 (NCT01673867)
				Malignant pleural mesotheliom	CHECKMATE-743 (NCT02899299)
				RCC	CHECKMATE-214 (NCT02231749) CHECKMATE-9ER (NCT03141177) CHECKMATE-025 (NCT01668784)
				cHL	CHECKMATE-039(NCT01592370)
				SCCHN	CHECKMATE-141(NCT02105636)
				Urothelial carcinoma	CHECKMATE-274 (NCT02632409) CHECKMATE-275(NCT02387996)
				Colorectal cancer	CHECKMATE-142 (NCT02060188)
				HCC	CHECKMATE -040 (NCT01658878)
				Esophageal cancer	CHECKMATE-577 (NCT02743494) CHECKMATE-648 (NCT03143153) CHECKMATE-649 (NCT02872116) ATTRACTION-3 (NCT02569242)
Cemiplimab(Libtayo)	Regeneron Pharmaceuticals	PD-1	A recombinant human IgG4 monoclonal antibody	CSCC	Study 1423 (NCT02383212) Study 1540 (NCT02760498)
				Basal cell carcinoma	Study 1620 (NCT03132636)
				NSCLC	Study 1624 (NCT03088540)
Dostarlimab (Jemperli)	GlaxoSmithKline	PD-1	An investigational humanized IgG4 monoclonal antibody	Endometrial cancer	GARNET (NCT02715284)
				solid tumors	GARNET (NCT02715284),
Atezolizumab (Tecentriq)	Genentech	PD-L1	An Fc-engineered, humanized, non-glycosylated IgG1 kappa immunoglobulin	HCC	IMbrave150(NCT03434379)
				Urothelial carcinoma	IMvigor210 (NCT02108652) IMvigor130 (NCT02807636)
				NSCLC	IMvigor010 (NCT02486718) IMpower110 (NCT02409342) IMpower150 (NCT02366143) IMpower130 (NCT02367781) OAK(NCT02008227)
				SCLC	IMpower133(NCT02763579)
				Melanoma	IMspire150 (NCT02908672)
Durvalumab (Imfinzi)	AstraZeneca	PD-L1	A human IgG1 kappa monoclonal antibody	SCLC	CASPIAN(NCT03043872)
				NSCLC	PACIFIC (NCT02125461)
				Urothelial carcinoma	Study1108(NCT01693562)
Avelumab(Bavencio)	EMD Serono	PD-L1	A human IgG1 lambda monoclonal antibody	RCC	JAVELIN Renal 101(NCT02684006)
				Urothelial carcinoma	JAVELIN Solid Tumor (NCT01772004)
				MCC	JAVELIN Merkel 200 (NCT02155647)
Ipilimumab (Yervoy)	Bristol-Myers Squibb	CTL-4	An IgG1 kappa immunoglobulin	HCC	CHECKMATE-040 (NCT01658878)
				Colorectal cancer	CHECKMATE-142 (NCT02060188)
				RCC	CHECKMATE-214 (NCT02231749)
				Melanoma	MDX010-20 (NCT00094653)
				Esophageal squamous cell carcinoma	CHECKMATE-648 (NCT03143153)
				Malignant pleural mesothelioma	CHECKMATE-743 (NCT02899299)
				NSCLC	CHECKMATE-9LA (NCT03215706) CHECKMATE-227 (NCT02477826)
Relatlimab-rmbw (Opdualag)	Bristol-Myers Squibb	LAG-3	A human IgG4 monoclonal antibody	Melanoma	RELATIVITY-047 (NCT03470922)

Prepared using data from FDA.gov

In contrast to primary resistance, acquired resistance is not clearly characterized across tumor types as its incidence has not been routinely reported [25]. Moreover, our understanding of acquired resistance is far from adequate and there is a desperate need to understand the mechanisms involving acquired resistance to determine the most optimal path forward for treating patients.

## Potential mechanisms underlying acquired resistance to immune checkpoint inhibitors

The mechanisms of acquired resistance are indistinguishable and interdependent, and seem to overlap at least partially with the mechanisms of primary resistance. Pseudo-progression that occurs in the initial or late stage of treatment complicates the identification of resistance mechanisms. In light of Darwinian natural selection, the resistance to ICIs therapy may be pre-existing in the tumor cells before treatment, and is subsequently acquired as a result of the process of immune selection due to the genomic and epigenomic instability of tumor cells [7]. Acquired resistance may also occur at the individual tumor cell level because of the alternation in gene expression of tumor cells in response to interactions with immune cells or their products [7, 26].

**Fig. 2:**
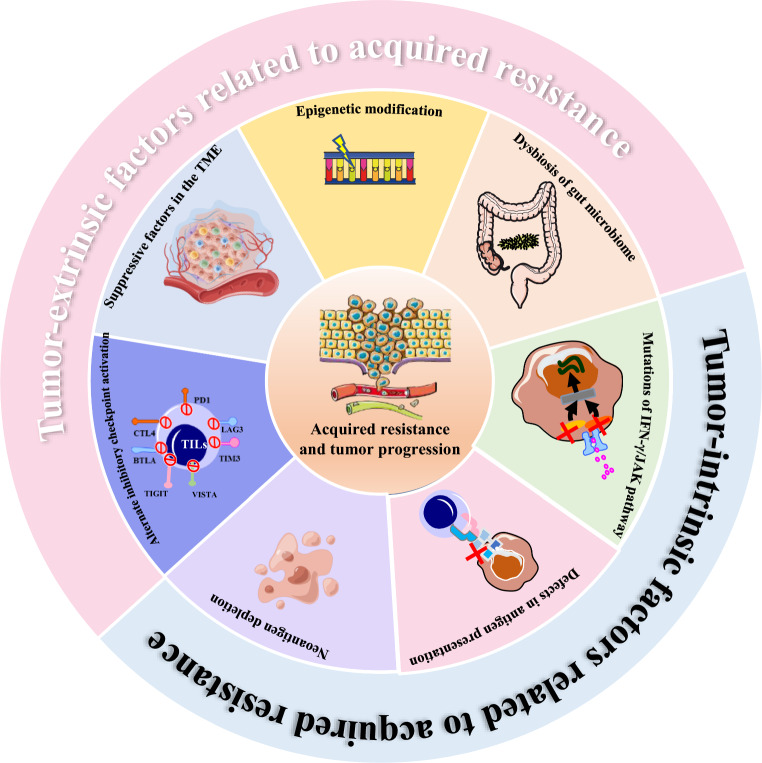
Graphical summary that explains underlying mechanism of acquired resistance to ICIs in terms of tumor cell-intrinsic and tumor cell-extrinsic factors. Tumor intrinsic mechanisms of acquired resistance involve loss of immunogenic antigens, defects in antigen processing and presentation, and mutations of IFN‐γ/JAK signaling pathway. Tumor extrinsic mechanisms of acquired resistance mainly include activation of alternate inhibitory immune checkpoints, immunosuppressive tumor microenvironment, epigenetic modification, and dysbiosis of the gut microbiome

Furthermore, the mechanisms leading to resistance might vary not only by tumor types but also by patient-specific factors due to the unique genetic and clinical backgrounds of each patient. Therefore, in this section, we have shed light on the current understanding of the mechanisms concerning acquired resistance in terms of tumor cell-intrinsic and cell-extrinsic factors, based on pre-clinical animal models and clinical trials (Fig. 2).

## Tumor-intrinsic factors related to acquired resistance

### Loss of immunogenic neoantigens

**Fig. 3:**
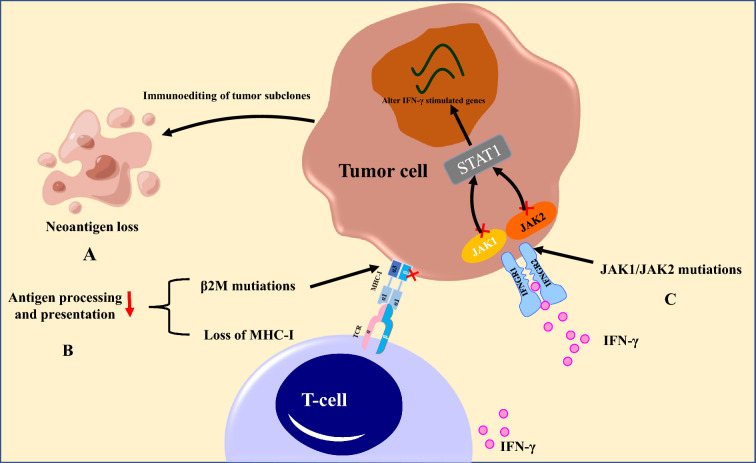
Tumor cell-intrinsic factors contributing to acquired resistance to ICI therapy. A: Neoantigens are in partial or total eliminated conferring poor immunogenicity and acquired resistance to ICIs therapy. B: β2M mutations affect the presentation of peptide MHC-I complexes to T cells, thus leading to the tumor cells not being recognized by T cells. C: Mutations of JAK1 and JAK2 make the tumor cells insensitive to IFN-γ secreted by T-cells

Neoantigens derived from somatic tumor non-synonymous mutations, which can be recognized by the immune system as “non-self”, are likely to be targets of tumor-specific T cells and elicit effective anti-tumor immune responses [21]. Tumor neoantigen burden is closely related to the immunogenicity and sensitivity to ICIs therapy [27]. Besides, tumors enriched with neoantigens have more abundant tumor-infiltrating cells (TILs) and higher levels of perforin mRNA and granzyme A, which is consistent with increased T-cell-mediated cytolytic activity [28]. Thus, mechanisms causing the loss of neoantigen expression during immunotherapy may lead to acquired resistance (Fig. 3). It has been proposed that cancer immunoediting comprises three sequential stages of elimination, equilibrium, and escape, which describe dynamic interactions between tumor cells and T cells [29]. Cancer immunoediting can lead to the partial or total elimination of neoantigens and the selection of subclones lacking neoantigen expression within the tumor, thereby conferring poor immunogenicity and acquired resistance to ICIs [29, 30]. A recent study by Anagnostou and colleagues was the first to demonstrate that acquired resistance to ICI therapy was related to the loss of mutations encoding putative mutation-associated neoantigens (MANAs) by eliminating tumor subclones or deleting chromosomal regions [31]. Specifically, whole-exome sequencing of matched resistant and pre-treatment tumors identified genomic alterations leading to the loss of 7 to 18 putative MANAs in resistant tumor clones from NSCLC patients who relapsed after an initial response to ICIs [31]. Acquired new mutations did not encode neoantigens, and the eliminated MANAs had higher predicted MHC binding affinity than either those that were gained or retained in the resistant tumors [31]. George et al. [32] identified the decreased expression of two neoantigens and loss of biallelic PTEN as the potential mechanisms of acquired resistance to ICI therapy by whole transcriptome analyses of pre-treatment and resistant tumor samples obtained from a uterine leiomyosarcoma patient. Moreover, it is arguable that the epitope of the CD19 protein sequence recognized by the chimeric antigen receptor may be selectively deleted at progression in some patients with acute lymphoblastic leukemia who demonstrated an initial response to CD19 adoptive T-cell therapy (ACT), and the pre-existing CD19 isoforms with alternative splicing may be prone to acquired resistance [33, 34].

The pro-inflammatory cytokines produced by TILs may induce the loss of neoantigen expression contributing to acquired resistance [35]. An ACT-treated mouse model exhibited transient tumor response following the injection of T cells targeting the melanoma differentiation antigen gp100. Subsequently, inflammatory cytokine interferon-γ (IFN-γ) secreted by TILs triggered a process of melanoma cell de-differentiation akin to epithelial-to-mesenchymal transition (EMT), leading to adaptation of melanoma cells by reducing gp100 expression and switching to a less differentiated neural crest phenotype [36]. Other inflammatory cytokines like transforming growth factor-β (TGF-β) and interleukin-6 (IL-6) have been shown to mediate the neuroendocrine differentiation of numerous types of histological cancers like lung and prostate cancer by EMT, resulting in immune escape and acquired resistance [35, 37, 38].

### Defects in antigen processing and presentation

Neoantigens must be processed by APCs and presented to T cells in the form of peptide MHC-I complexes [39]. Thus, a decrease in mRNA transcription of MHC molecules, loss of genome, as well as mutations of the β-2-microglobulin (β2M) gene negatively affect antigen presentation and contribute to resistance to ICIs therapy [40] (Fig. 3). Paulson et al. [41] found a transcriptional loss of genes encoding MHC-I in two Merkel cell carcinoma patients whose tumors had relapsed after initial response to PD-1/PD-L1 inhibitors. Zaretsky and coworkers reported that acquired resistance to anti-PD-1/PD-L1 therapy was associated with a truncating mutation of the gene encoding β2M, which was related to MHC-I, and essential for its folding and transport to the surface of T cells [42, 43]. Immunohistochemical analysis of the MHC-I heavy chains in tumor samples obtained from the patient revealed that adventitial localization was lost when compared to pre-treatment tumor and adjacent stroma, despite continued production of MHC-I molecules in the recurrent tumor [43]. β2M aberrations including multiple early frameshift mutations, absence of tumor-specific β2M protein expression, and loss of heterozygosity overlapping β2M were reported to occur in about 30% of melanoma patients with progressive disease after anti-PD-1 therapy, leading to a total loss of MHC-I [44]. Knock-out of β2M in an immunocompetent lung cancer mouse model conferred resistance to PD-1 blockade in vivo*,* proving its role in the resistance to ICIs [40]. Tran et al. [45] described the direct loss of the chromosome 6 haplotype encoding the HLA-C*08:02 in a patient with metastatic colorectal cancer that progressed after initial response to therapy consisting of HLA-C*08:02-restricted TILs targeting mutant KRAS G12D. Other mechanisms involved in the loss of MHC-I molecules have been reported, including downregulation of the transporter associated with antigen processing 2 and low‐molecular‐weight protein 7 in MSI‐negative colorectal tumors [46]. Furthermore, the increase in PD-1^+^ T cell infiltration is significantly related to the increase in β2M mutations, which indicates that the resistance induced by β2M mutations is associated with PD-1^+^ T cell infiltration.

## Mutations of IFN‐γ/JAK signaling pathway

Mutations of the janus kinases 1 and 2 (JAK1/JAK2) in the downstream signaling pathway of IFN-γ are emerging as pivotal factors in acquired resistance to ICIs therapy (Fig. 3). IFN-γ secreted by tumor-specific T cells binds to the heterodimeric IFNGR1/IFNGR2 receptor complex on tumor cells and causes the activation of JAK1 and JAK2, which in turn phosphorylate a transcription factor known as signal transducer and activator of transcription (STAT) 1. Subsequently, the translocation of phosphorylated STAT1 homodimers to the nucleus modulates the transcription of IFN-γ stimulated genes, which increases the production of chemokines, thereby attracting immune cells and directly promoting tumor cell apoptosis [47, 48]. Additionally, IFN‐γ/JAK signaling can also induce proteasome subunits and transporters associated with MHC-I as well as upregulate expression of PD-L1, which enhances antigen presentation and response to PD-1 antibodies [49, 50]. Whole-exome sequencing of tumor tissues obtained at baseline (before therapy) and after disease progression from four melanoma patients who displayed disease progression after a median 1.8 years objective response to pembrolizumab, revealed that two of the patients exhibited over 90% truncating dysfunctional mutations in JAK1 (Q503* nonsense mutation) and JAK2 (F547 splice‐site mutation), concurrent with the deletion of the wild-type allele and duplication of the mutant allele [43]. The deactivation of JAK1 and JAK2 signaling led to acquired resistance to IFN‐γ, subsequently causing damage to immune surveillance and tumor cell proliferation [43]. Several studies have shown that the loss of JAK/STAT signaling leads to resistance to CTLA-4 and PD-1 inhibitors because of the inability to upregulate PD-L1 and MHC-I expression [48, 49, 51]. Of concern, loss-of-function mutations existing at high frequency in tumors may be associated with primary resistance to ICIs therapy, with pretreatment melanoma biopsies demonstrating IFN‐γ pathway mutations in as many as 19% of the samples [48, 52]. Mutations in this pathway could additionally lead to the loss of PD-L1 expression upon IFN-γ exposure, thereby causing resistance to inhibitors of PD-1/PD-L [48]. Mutations and deletions of IFN‐γ pathway-associated proteins, like IFNGR1 and IFNGR2, as well as STAT1, may also be a reason for resistance to ICIs and need to be further explored [52].

## Tumor-extrinsic factors related to resistance

### Activation of alternate inhibitory immune checkpoints

**Fig. 4:**
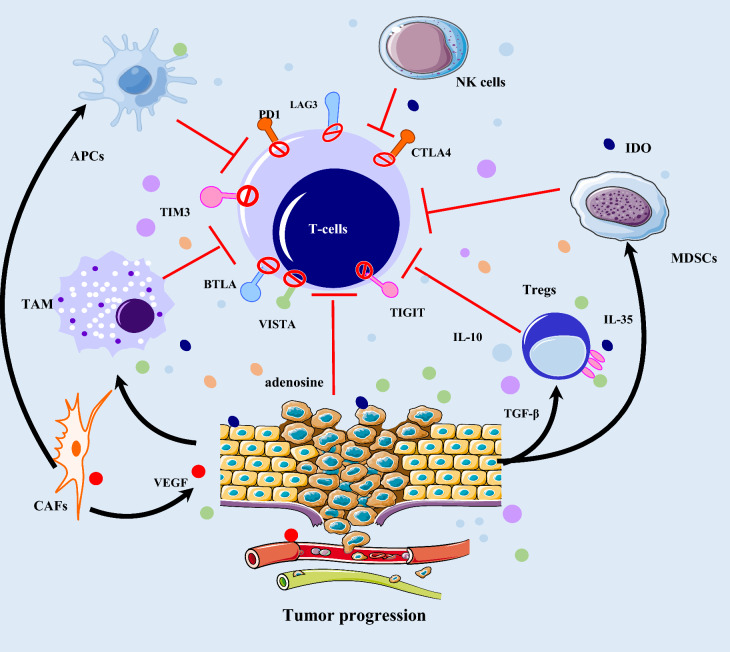
Immunosuppressive tumor microenvironment contributing to acquired resistance to ICI therapy. Immune cells infiltrate into the TME, interacting with each other and tumor cells, harbor an immunosuppressive phenotype responsible for immune escape of tumor cells and the following acquired resistance during or after ICI treatment. These immunosuppressive cells include Tregs, TAMs, MDSCs, which express the alternate inhibitory immune checkpoints like TIM-3, LAG-3, BTLA, VISTA, and TIGIT, secrete cytokines and growth factors like IL-35, IL-10, and TGF-β, VEGF, or product IDO and adenosine, which negatively regulate anti-tumor immune immunity, remodel the extracellular matrix, and promote angiogenesis. As a result, the immunosuppressive TME promotes the tumor cell resistance to ICI therapy

Overexpression of multiple alternate immune checkpoints contributing to a severely exhausted phenotype of T-cells can exert immunosuppressive effects and lead to the failure of ICI therapy. Acquired resistance to ICIs has been described secondary to compensatory upregulation of alternative immune checkpoints like T cell immunoglobulin and mucin domain 3 (TIM-3), LAG-3, B and T lymphocyte attenuator (BTLA), V-domain Ig suppressor of T cell activation (VISTA), T cell Ig and ITIM domain (TIGIT), and so on in multiple studies (Fig. 4). A study by Thommen et al. [53] revealed that increased coexpression of CTLA-4, PD-1, TIM-3, LAG-3 as well as BTLA, was positively related to progressive T-cell exhaustion and subsequent resistance to anti-PD1 therapy in NSCLC. Their research suggested that the surface of CD8^+^ T cells expressing the above five receptors had serious defects causing reduced proliferation, migration, and cytokine production. Koyama et al. [54] observed that TIM-3 up-regulation in TILs of lung adenocarcinoma patients was significantly correlated with acquired resistance to anti-PD-1 antibody. Moreover, the TIM-3 expression was related to the degree of anti-PD-1 antibody binding in T cells, the positive expression of which increased with the binding degree of T cells to anti-PD-1 blockade [54]. T-cells from resistant tumors also over-expressed LAG3 and CTLA4, however, BTLA expression appeared variable. Limagne et al. [55] demonstrated that the accumulation of monocytes, bone marrow-derived suppressor cells (MDSCs), and lymphocytes expressing TIM-3 and galectin-9 was related to primary or acquired resistance following nivolumab treatment in a cohort of NSCLC patients. Compared to matched pre-treatment tumors, the frequency of VISTA expression on T cells and CD68^+^ macrophages were found higher in treated prostate and melanoma tumors [56]. Kakavand et al. found that twelve melanoma patients with acquired resistance to ICIs were able to obtain matched samples before and after treatment, 8 of which patients were detected with VISTA upregulation [57]. Several studies suggested that the high expression of TIGIT regulatory T cells (Tregs) positively related to increased Treg frequencies in tumors and mediate tumor resistance to ICI therapy [58, 59]. The upregulation of other immune checkpoints also potentially results in acquired resistance that needs further research.

### Other suppressive factors in the tumor microenvironment (TME)

It is now increasingly appreciated that immunosuppressive cells including Tregs, MDSCs, tumor-associated macrophages (TAMs), and cancer-associated fibroblasts (CAFs), as well as some inhibitory cytokines, build a chronic inflammatory, pro-angiogenic, intratumoral, and immunosuppressive environment where tumor cells are liable to evade immune-mediated eradication and inhibit the therapeutic activity of ICIs [60–62] (Fig. 2). Tregs may be capable of suppressing the efficacy of effector T cells (TEFFs) by the secretion of some inhibitory cytokines like TGF-β, IL-10, and IL-35 [63, 64]. The response to PD-1/PD-L1 inhibitors has been shown to be correlated with an increased ratio of TEFFs to Tregs in murine models [65]. Simpson et al. [66] suggested that an increased Tregs infiltration resulted in acquired resistance to PD-1 inhibitors alone or in combination with radiotherapy. ICI treatment might cause the recruitment of Tregs to the TME and play a role in mediating resistance, as demonstrated in a murine model of claudin-low breast cancer generally known to be resistant to ICIs [67]. Timelines from clinical studies indicate that increased MDSCs in the TME promoting tumor invasion, angiogenesis, and metastasis correlate with the reduced efficacy of immunotherapies including ICIs [68, 69]. Previous clinical studies have revealed the relationship between increased infiltration of TAMs and ominous prognosis [31, 70]. TAMs can express co-inhibitory molecules, including PD-L1 and B7-H4, and release anti-inflammatory cytokines, such as IL-10 [71]. M2 macrophages were shown to directly remove anti-PD1 antibodies from the surface of PD-1^+^ T cells, thereby interfering with anti-PD1 therapy [70]. Thought to be one of the most abundant components in the mesenchyme of most tumors, CAFs contribute to T-cell dysfunction and immunosuppressive TME by producing several CAF-derived molecules and ligands, including IL-6, chemokine C-X-C motif ligand 12 (CXCL12), CXCL5, TGF-β, vascular endothelial growth factor (VEGF), fibronectin, and collagen, and the increased expression of immune checkpoint ligands including PD-L1, PD-L2, and FasL [72]. CAFs also promote a Dendritic cell (DC) phenotype that is unable to interact with and present antigens to CTLs [73]. Chakravarthy et al. identified a poor prognosis phenotype of CAFs driven mainly by the activation of TGF-β signaling, which was related to the PD-1 blockade resistance in bladder cancer and melanoma [74]. Increased infiltration of CAFs was related to resistance to PD-1 blockade, inhibition of which rescued the anti-tumor effects of anti-PD-1 treatment in mouse models of hepatocellular carcinoma [75]. Indole 2,3-dioxygenase (IDO), secreted by immune cells or tumor cells, can promote the secretion and activity of Tregs and MDSCs [76]. As a rate-limiting enzyme, IDO can promote the catabolism of tryptophan and generate immunosuppressive metabolites including kynurenine, thereby suppressing the function of T cells and inducing T cell apoptosis [77, 78]. Holmgaard et al. [79] reported that tumor growth was significantly delayed and OS markedly increased in IDO knockout melanoma mice treated with anti-PD1 therapy compared with that in wild-type mice. Also, the accumulation of extracellular adenosine though the HIF-1α-CD39/CD73-adenosine signaling pathway has been proven to drive an immunosuppressive TME, and promote tumor progression [80].

## Influence of epigenetic modification

**Fig. 5:**
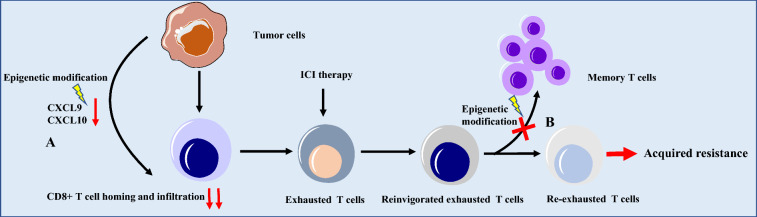
Epigenetic modification and acquired resistance to ICI therapy. A: Epigenetic silencing of gene of T helper 1-type chemokines CXCL9 and CXCL10 results in CD8+ T cell homing and infiltration to the TME. B: Acquisition of memory potential was limited after reinvigoration of exhausted T cells with PD-L1 blockade. Reinvigorated exhausted T cells fail to become memory T cells after antigens clearance and they will become depleted again, even if neoantigens are exposed again

Epigenetic modification of tumor cells has been shown to alter the expression of immune-associated genes, thus affecting the processing and presentation of antigens, proliferation, and differentiation of T cells, T-cell exhaustion, and acquisition of phenotype of memory T cells [81] (Fig. 5). Lower levels of IFN -γ and IFN-γ-induced genes like CXCL9 contribute to decreased infiltration of CD8+ T cells and are correlated with tumor progression following anti-PD-L1 therapy [82]. A study by Peng et al. [83] revealed that cancer epigenetic silencing consisting of enhancer of zeste homolog 2 (EZH2)-mediated histone modifications and DNA methyltransferase (DNMT) -mediated DNA methylation of the gene encoding the T helper 1-type chemokines, CXCL9 and CXCL10, decreased T cells homing and infiltration, thus potentially impairing the efficacy of immunotherapy. Recent studies have suggested that the exhaustion of T cells correlated with de novo DNA methylation and may persist and be passed on to successive T cell generations [84]. Epigenetic studies have suggested that although ICIs can reinvigorate exhausted CD8^+^ T cells (TEX) and enhance the control of chronic infections and tumors, they have little effect on genes related to the generation of memory T-cells [85]. They found that TEX metagenes treated with PD-L1 blockade partly overlapped with TEFF, but there was little overlap with memory T cells (TMEM), indicating that acquisition of memory potential was limited after TEX reinvigoration [85]. Reinvigorated TEX failed to become TMEM after antigen clearance and they became depleted again following anti-PD-L1 treatment, even if there was neoantigens re-exposure. Although the inhibition of the PD-1 pathway led to transcriptional re-wiring and re-engagement of effector circuitry in the TEX epigenetic landscape, TEX could never acquire the phenotype of memory T cells, and this epigenetic stability of TEX might result in acquired resistance to ICIs [85]. In addition, TEX showed extensive changes in the available chromatin, which were different from those in the TEFF counterparts. Exhaustion-specific enhancers in TEX displayed different motifs at T-bet, RAR, and Sox3 marker transcription factor binding sites. The crucial differences in the profile of regulatory regions between functional CD8+ T cells and TEX were greater than those observed in gene expression, which suggests that the accessible chromatin networks related to the exhaustion state required a large rewiring [86]. A combination of ICIs and epigenetic modifiers may help to improve the durability of the response to ICIs in the future.

### Dysbiosis of gut microbiome

It is emerging that the alteration of the human gut microbiome affects tumor progression and is connected with the response to ICIs therapy [87]. Significant differences in the structure of gut microbiome were observed between patients who responded well to ICIs and those who did not [88, 89]. A landmark study conducted by Gopalakrishnan et al. demonstrated that patients who have a high diversity and abundance of gut microbiota like *Faecalibacterium* and *Ruminococcaceae* have increased antigen presentation and improved TEFF function in the periphery and TME with enhanced anti-tumor immune response, whereas patients with low diversity and high relative abundance of gut microbiota like *Bacteroidales* have impaired anti-tumor immune responses mediated by a decreased capacity of antigen presentation and limited intratumoral lymphoid and myeloid infiltration [90]. G Analysis of the gut microbiome composition of patients with metastatic melanoma before anti-PD-1 treatment indicated that bacterial species including *Ruminococcus obeum* and *Roseburia intestinalis* were remarkably abundant in non-responders, while *Enterococcus faecium*, *Collinsella aerofaciens*, and *Bifidobacterium longum* were significantly enriched in responders [91]. A prospective study that enrolled 26 metastatic melanoma patients treated with ipilimumab found that antitumor response and immune-related colitis were related to colonization by Firmicutes including butyrate-producing bacterium L2–21, *Faecalibacterium prausnitzii* L2–6, and *Gemmiger formicilis* ATCC 27749. Compared to patients with initial microbiota enriched with Bacteroidetes, patients with microbiota rich in *Faecalibacterium* genus and other Firmicutes had a markedly higher progression-free survival (PFS) and OS [92]. Antibiotic use and gastrointestinal immune-related adverse events (irAEs), which are common complications of ICIs therapy, disrupt the intestinal microbiome and result in acquired resistance [93]. Pre-clinical mouse models have indicated that broad-spectrum antibiotics like imipenem and colistin eliminated gut microbiota and decreased the antitumor activity of CTLA-4 antibodies, with a significant reduction in the anti-CTLA-4-mediated sensitization of tumor-infiltrating and splenic lymphocytes. *Bacteroides fragilis* could recover the response to CTLA-4 antibodies, while tolerogenic *Bacteroides* species mediated complete resistance to CTLA-4 antibodies [94]. Also, clinical data from a large cohort of patients with non-small cell lung cancer, renal cell carcinoma, or urothelial cancer suggested that patients treated with antibiotics for routine indications shortly before, during, or shortly after treatment with anti-PD1/PD-L1 mAB had significantly lower PFS and OS rates compared to patients who had not received antibiotics, suggesting that disrupted the gut microbiota via antibiotic use could potentially impair anti-tumor immune responses and resulted in acquired resistance to ICIs [89]. The impact of gut microbiome alteration further increases the complexity of responsiveness and resistance to ICIs therapy. Large prospective trials are required to define the microbial components and mechanisms underlying this effect, and to provide a basis for introducing different kinds of gut bacteria to reverse immunotherapy resistance in cancer.

### Potential strategies to overcome resistance: combining immune checkpoint therapy with other therapies

The above-mentioned mechanisms of acquired resistance to ICIs do not act in isolation, and the interactions between several immunosuppressive components and the overlap between various signaling pathways together resulted in resistance. Identifying the specific resistance mechanisms in these patients is a crucial step toward effective treatment and ultimately inducing durable responses for them. It is likely that combination therapies with multiple treatment modalities have potential synergistic effects and are likely to reverse resistance. It is worth noting that efforts should be guided by an understanding of the mechanisms underpinning resistance rather than the random combination with existing drugs and therapies. In this section, we discussed potential approaches to counter the resistance and improve the efficacy of ICIs from the perspective of the resistant mechanisms mentioned above (Table 2).

**Table 2:** Summary of agents with potential to overcome acquired resistance to immune checkpoint inhibitors

Category	Agent	Target	combinational therapies	Tumor type (s)	Phase	References
Targeting neoantigen depletion	Neo-PV-01	Personalized neoantigen-based vaccine	Nivolumab	Melanoma NSCLC Bladder Cancer	Phase Ib (NCT02897765)	[98]
	ISA 101	Synthetic long-peptide HPV-16 vaccine	Nivolumab	HPV-16-positive cancer	Phase II (NCT02426892)	[99]
	T-VEC	Oncolytic virus	Pembrolizumab	Melanoma	Phase Ib (NCT02263508)	[102]
	T-VEC	Oncolytic virus	Ipilimumab	Melanoma	Phase II (NCT01740297)	[103]
	T-VEC	Oncolytic virus	Ipilimumab; Nivolumab; Pembrolizumab	Melanoma	Retrospective	[104]
Targeting defects in antigen processing and presentation	LCL161	The cIAP1/2 antagonist	Anti-PD-L1 and/or anti-CTLA4	Pancreatic ductal adenocarcinoma	Preclinical	
	Trametinib	The mall molecule MEK inhibitor	Anti-PD-L1	HNSCC	Preclinical	[106]
	Chloroquine	Inhibiting autophagy	Anti-PD1 and anti-CTLA4	PDAC	Preclinical	[107]
	ASPIRE nanovaccine	Specific pMHC-I, anti-PD1 antibody and B7 are simultaneously anchored by a programmed process	NA	Melanoma Colon carcinoma Lewis lung carcinoma	Preclinical	[108]
Targeting IFN‐γ/JAK signaling pathway	BO-112	A nanoplexed formulation of Poly I: C coupled to polyethylenimine	Anti-PD-L1	Melanoma	Preclinical	[110]
	BO-112	A nanoplexed formulation of Poly I: C coupled to polyethylenimine	Pembrolizumab; Nivolumab	Solid tumor	Phase I (NCT02828098)	[111]
	Overexpression of NLRC5	Upexpressing NLRC5	ACT	Melanoma	Preclinical	[112]
Targeting alternate ICI activation	F38-2E2	Anti-TIM-3 antibody	Nivolumab	Lung cancer	Preclinical	[55]
	Ieramilimab	The LAG-3 inhibitor	Spartalizumab	Advanced malignancies	Phase I/II (NCT02460224)	[115]
	MIH63	Anti-VISTA antibody	Anti-PD-1 and anti-CTLA-4	Squamous cell carcinoma	Preclinical	[116]
	Anti-murine BTLA antibody	Anti-BTLA antibody	Anti-PD-1	Glioblastoma	Preclinical	[117]
	Tiragolumab	Anti-TIGIT antibody	Atezolizumab	NSCLC	Phase II (NCT03563716)	[118]
Targeting suppressive tumor microenvironment	anti-CD25 antibody	Anti-CD25 antibody	Anti-PD-1	Sarcoma, colon adenocarcinoma, melanoma, and colorectal carcinoma	Preclinical	[119]
	Mogamulizumab	Anti-CCR4 antibody	Nivolumab	Solid tumors	Phase I (NCT02476123)	[121]
	PLX3397	CSF-1R kinase inhibitor	Oncolytic viruses, and anti-PD-1	Colon cancer	Preclinical	[123]
	BLZ945	CSF-1R kinase inhibitor	Anti-PD-1 and anti-PD-L1	Control spontaneous neuroblastoma	Preclinical	[124]
	ATRA	Targeting MDSCs	Anti-PD-1	NSCLC	Preclinical	[125]
	ATRA	Targeting MDSCs	Ipilimumab	Melanoma	Phase II (NCT02403778)	[126]
	BMS-211	Targeting MDSCs	anti-CTLA-4	Lung carcinoma, colon carcinoma, breast carcinoma and lymphoma	Preclinical	[127]
	Epacadostat	The IDO1 enzyme inhibitor	Pembrolizumab	Solid tumors	Phase I/II (NCT02178722)	[128]
	Epacadostat	The IDO1 enzyme inhibitor	Pembrolizumab	Melanoma	Phase III (NCT02752074)	[129]
	Linrodostat mesylate	The IDO1 enzyme inhibitor	Nivolumab	Bladder cancer	Phase I/II (NCT02658890)	[130]
	CD39 knockout	Blocking CD39	anti-PD1	HCC	Preclinical	[131]
	Oleclumab	Anti-CD73 monoclonal antibody	Durvalumab	NSCLC	Phase II (NCT05221840)	[132]
	NIR178	The A2AR antagonist	Immunotherapy	NSCLC	Phase II (NCT02403193)	[133]
	TGFβ-blocking	Inhibiting TGFβ	Anti-PD-L1	Urothelial cancer	Preclinical	[134]
	Mithramycin		Anti-PD-L1	Chronic lymphocytic leukemia	Preclinical	[135]
	Pegilodecakin	The PEGylated form of IL10	Pembrolizumab or nivolumab	Solid tumors	Phase IB (NCT02009449)	[136]
Targeting epigenetic modification	Decitabine	The DNMT inhibitor	anti-CTLA-4	Ovarian Cancer	Preclinical	[142]
	Azacitidine	The DNMT inhibitor	Nivolumab	Acute Myeloid Leukemia	Phase II (NCT02397720)	[148]
	Panobinostat	The HDAC inhibitor	Anti-PD-L1	Melanoma	Preclinical	[143]
	Romidepsin	The HDAC inhibitor	Anti-PD-L1	Lung Adenocarcinoma	Preclinical	[144]
	HBI-8000	The HDAC inhibitor	PD-1, PD-L1, or CTLA-4,	Colon carcinoma and B-cell lymphoma	Preclinical	[145]
	Vorinostat	The HDAC inhibitor	Pembrolizumab	NSCLC	Phase I/Ib (NCT02638090)	[149]
	Entinostat,	The HDAC inhibitor	Pembrolizumab	Melanoma	Phase Ib/II NCT02437136	[150]
	Entinostat	The HDAC inhibitor	Pembrolizumab	NSCLC	Phase Ib/II NCT02437136	[151]
	GSK503	The EZH2 inhibitor	Anti-PD1, anti-CTLA-4 and IL-2	Melanoma	Preclinical	[146]
Targeting gut microbiome Dysbiosis	*Bifidobacterium*	Regulating gut microbiota	Anti-PD-L1	Melanoma	Preclinical	[153]
	*Bacteroides fragilis*	Regulating gut microbiota	Anti-CTLA-4	Melanoma	Preclinical	[94]
	*Lactobacillus johnsonii*, *Bifidobacterium pseudolongum*, and *Olsenella*	Regulating gut microbiota	Anti-CTLA-4	Colorectal cancer, bladder cancer, and melanoma	Preclinical	[154]
	EDP1503	Regulating gut microbiota	Pembrolizumab	Solid tumors	Phase I/II (NCT03775850)	[155]
	MRx0518	Regulating gut microbiota	Pembrolizumab	Bladder cancer, NSCLC, RCC, and Melanoma	Phase I/II (NCT03637803)	[156]
	FMT	Regulating gut microbiota	Anti-PD-1	Epithelial tumors	Preclinical	[89]
	FMT	Regulating gut microbiota	Nivolumab	Melanoma	Phase I NCT03353402	[157]
	FMT	Regulating gut microbiota	Pembrolizumab	Melanoma	Phase II NCT03341143	[158]
Other emerging combination strategies	STING-LNP	Stimulating the STING pathway	Anti-PD-1	Melanoma	Preclinical	[159]
	MUSIC platform	Stimulating the STING pathway	Anti-PD-1	Breast cancer	Preclinical	[160]
	iTPNCs	Activating inflammatory signaling	Anti-CTLA-4	Melanoma, colon cancer, and breast cancer	Preclinical	[161]
	^177^Lu-PSMA-617	Delivering beta-particle radiation	Pembrolizumab	Prostate cancer	Phase II NCT03805594	[162]
	BNT111	RNA vaccine	Anti-PD-1	Melanoma	Preclinical	[163]
	8FNs	Nanovaccine	Anti-PD-1	Melanoma	Preclinical	[164]

## Targeting neoantigen depletion

It has been demonstrated that autologous tumor vaccinations enhancing immunogenicity and stimulating adaptive immune responses with tumor-specific antigens improved sensitivity to ICI therapy [95–97]. NEO-PV-01, a neoantigen-based vaccine, was tested combined with nivolumab in the phase Ib clinical trial (NCT02897765) in NSCLC, melanoma, and bladder cancer demonstrated that neoantigen-specific CD4^+^ and CD8^+^ T cell responses were detected in all vaccinated patients with no serious adverse events [98]. The combined therapy of the vaccine ISA 101 and nivolumab for patients with incurable HPV-16-positive tumors achieved an overall response rate of 33% and a median OS of 17.5 months, much higher than the results obtained with nivolumab monotherapy, rendering an overall response rate of 14.3% [99]. Guo et al. reported that neoantigen-loaded monocyte-derived dendritic cell vaccines in combination with nivolumab triggered neoantigen-specific CD4^+^ and CD8^+^ T cell activation and mediated complete regression of all tumors in advanced metastatic gastric cancer [100]. The combination of oncolytic virotherapy and ICI therapy is a promising approach. Similar to self vaccination, oncolytic virustherapy could also induce tumor antigen release and provide danger signals, which consequently led to enhanced T cell priming and ameliorated resistance to anti-PD1/PDL1 therapy [101]. Talimogene laherparepvec (T-VEC) has been proven to improve the efficacy of anti-PD-1 therapy in advanced melanoma patients, with an objective response rate of 62% and a complete response rate of 33% [102]. A phase II Study (NCT01740297) suggested that the combination of T-VEC and ipilimumab shows a higher objective response rate versus ipilimumab alone without additional safety concerns in patients with advanced unresectable melanoma [103]. Consistently, a retrospective study suggested that T-VEC treatment might overcome loco-regional ICI acquired resistance in patients with stage IIIB-IV M1c melanoma [104].

## Targeting defects in antigen processing and presentation

Growing evidence suggests that the correction of defects in antigen processing and presentation can greatly enhance the efficacy of ICI therapy. It was reported that the cellular inhibitor of apoptosis proteins 1 and 2 (cIAP1/2) antagonism controlled the growth of β2M deficient tumors, and was effective in models of either primary or acquired resistance to ICIs [105]. In a mouse model of MAPK-activated head and neck squamous cell carcinoma, trametinib, a small molecule MEK inhibitor, inhibited extracellular signal-regulated kinase phosphorylation, promoted MHC-I and PD-L1 expression, and in combination with PD-L1 blockade, overcame resistance to monotherapy, which significantly improved inhibition of tumor growth [106]. Results from a mouse model of pancreatic ductal adenocarcinoma showed that chloroquine enhanced MHC-I antigen expression, sensitizing tumors to dual immune checkpoint inhibition with anti-CTLA4 and anti-PD-1 antibodies [107]. The ASPIRE nanovaccine derived from recombinant adenovirus-infected dendritic cells, in which specific peptide-MHC-I, B7 co-stimulatory molecules, and anti-PD1 antibody were simultaneously anchored by a programmed process, could significantly enhance the delivery of antigen to lymphoid organs and induce broad-spectrum T-cell responses to eliminate established tumors [108]. Additionally, regimens targeting TLRs (e.g., TLR3, TLR9) have been shown to promote DC maturation and reasonably alleviate anti-PD1/PDL1 resistance [109].

## Targeting the IFN‐γ/JAK signaling pathway

In experiments in mice, intratumoral BO-112 (a nanoplexed formulation of Poly I: C coupled to polyethylenimine) exerted anti-tumor activities in an IFN-γ and IFN-α/β dependent manner, which was observed not only in locally injected tumors but also in distant tumors. And the systemic effect was augmented by the coadministration of anti–PD-L1 antibodies [110]. A multicenter phase I clinical trial (NCT02828098) incorporating 28 patients with tumors resistance to anti–PD-1 antibodies showed the combination of BO-112 administered intratumorally with pembrolizumab or nivolumab was also well-tolerated, with 3 patients achieved partial responses, with 10 more patients having stable disease, suggesting that local BO-112 might be a strategy to revert anti-PD-1 therapy [111]. ACT was effective against JAK2 loss tumors, but not JAK1 loss tumors in an IFN signaling-deficient model of B16 murine melanoma, and overexpression of NLRC5(nucleotide-binding oligomerization domain-like receptor family caspase recruitment domain containing 5) restored the efficacy of ACT against B16-JAK1 loss tumors [112].

## Targeting activation of alternate inhibitory immune checkpoints

The combination therapies of ICIs targeting different immune checkpoints indicate potential benefits in overcoming acquired resistance in pre-clinical studies and clinical trials. The combination of anti-CTLA-4 and anti-PD-1 treatment appears to target separate populations of T-cells and has already been approved in hepatocellular carcinoma, melanoma, renal cell carcinoma, and colorectal cancer, with a variety of trials ongoing [113, 114]. Limagne et.al suggested that TIM-3 blockade could restore in vitro the CD8 secreting property in PBMCs from lung cancer patients when used in combination with the anti-PD-1 antibody, suggesting that anti-TIM-3 reverses resistance to anti-PD-1 [55]. Phase I/II study (NCT02460224) suggested that the LAG-3 inhibitor ieramilimab plus anti-PD-1 spartalizumab were well tolerated and had anti-tumor activity, with elicited durable responses in 12 of 121 patients with solid malignancies [115]. Co-blockading PD-L1and VISTA in CT26 colon cancer and B16 melanoma mouse models showed better efficacy in inhibiting tumor growth and prolonging survival compared to targeting each molecule alone, suggesting a synergistic effect between anti-VISTA and anti-PD-L1 antibodies in inducing Teff activation [116]. Choi et al. showed that the combination of PD-1 blockade and BTLA inhibitier synergistically improved overall long-term survival compared to monotherapy, with decreased levels of Tregs and increased expression of CD4^+^ IFN-γ and CD8^+^ IFN-γ [117]. Interim data from a phase II trial (NCT03563716) demonstrated the improved efficacy of Dual inhibition of TIGIT and PD-L1 with tiragolumab and atezolizumab compared to atezolizumab alone in patients with PD-L1-positive metastatic NSCLC in terms of overall response and progression free survival without additional adverse events [118].

## Targeting suppressive factors in the tumor microenvironment

It seems that immunosuppress components within the TME represent promising targets for increasing the anti-tumor efficacy of immunotherapy. A pre-clinical study showed that anti-CD25 antibody could effectively deplete tumor-infiltrating Tregs and increase ratios of effector-to-Treg, and synergized with PD-1 blockade to promote complete tumor rejection [119]. It has been suggested that CC chemokine receptor 4 (CCR4) mediated Treg recruitment into the TME, antagonism of which could suppress the frequency of CCR4 reduced Tregs and potentiated anti-tumor efficacy of ICIs [120]. A phase I study (NCT02476123) showed that combining nivolumab, with mogamulizumab (a Treg-depleting anti-CCR4 antibody) provided an acceptable safety profile and anti-tumor activity, with populations of effector Tregs reduced and CD8^+^ T cells in TILs increased [121]. In the tumor TME, TAMs survival depends on the CSF1/CSF1R pathway, blocking CSF1/CSF1R has been proven to significantly reduce macrophage recruitment and M2 phenotype polarization and activates CD8^+^ T cells, thereby sensitizing tumor to ICI and prolonged survival in pancreatic ductal adenocarcinoma and hepatocellular carcinoma [122]. Results according to mouse models of colon cancer revealed that CSF-1R kinase inhibitor PLX3397 combined with anti-PD-1 antibody and oncolytic viruses synergistically conferred significant tumor control and prolonged the survival [123]. Antagonizing CSF-1R with BLZ945 could reduce the induction of human and murine suppressive myeloid cells, when combined with PD-(L)1 blockades superiorly limited tumor progression [124]. Due to the function of modulating the differentiation of MDSCs, the combinational therapies of ATRA and immunotherapies have synergistic anti-tumor efficacy. Depleting MDSCs by all-trans-retinoic acid (ATRA) in the model of LKB1-deficient murine tumors resulted in improved anti-tumor T cell responses and increased sensitivity to PD-1 blockade [125]. Randomized phase II clinical trial (NCT02403778) reported that ATRA plus ipilimumab significantly decreased the number of MDSCs in the peripheral circulation of patients with metastatic melanoma without increased incidence of grade 3 or 4 adverse events, compared with ipilimumab monotherapy [126]. Besides, inhibition of casein kinase 2 (CK2) with BMS-211 substantially reduced the amount of polymorphonuclear MDSCs and macrophage differentiation, which was dramatically synergized with the anti-tumor efficacy of CTLA-4 blockades [127]. The phase I/II ECHO-202/KEYNOTE-037 trial (NCT02178722) revealed that epacadostat (a selective IDO1 enzyme inhibitor) plus pembrolizumab generally had a manageable safety profile and encouraging anti-tumor activity in several advanced solid tumors like melanoma, NSCLC, squamous cell carcinoma of the head and neck, renal cell carcinoma, urothelial carcinoma, and endometrial adenocarcinoma [128]. Nevertheless, epacadostat had no additional clinical benefit to pembrolizumab in the pivotal phase III study [129]. A Phase I/II trial (NCT02658890) suggested that linrodostat mesylate (an oral IDO1 inhibitor) in combination with nivolumab has demonstrated safety and preliminary evidence of clinical activity, with an ORR of 34% in the advanced bladder cancer [130]. Several preclinical studies have implicated that unleashing the immunosuppressive TME by targeting CD39/CD73 (that generate adenosine) and anti- adenosine agents (that consume adenosine) could suppress acquired resistance thus synergy ICIs efficacy [121]. Lu et.al has demonstrated that targeting CD39 on macrophages could efficiently rescue anti-PD1 resistance in hepatocellular carcinoma (HCC) [131]. The phase II trial of Oleclumab targeting CD73 in combination with Durvalumab showed prolonged PFS and increased ORR versus durvalumab alone, with manageable safety profiles [132]. Results from the phase I/II study of the A2AR antagonist NIR178 in advanced NSCLC suggested that clinical benefit was observed in immunotherapy-exposed and -naïve patients regardless of PD-L1 status, and was well tolerated [133]. A number of such combination therapies are currently ongoing in clinical trials. Moreover, vascular normalization with VEGF/VEGFR inhibitors combined with PD-(L)1 blockade has demonstrated clinical efficacy across a variety of tumor types in phase III trials and is already FDA-approved in NSCLC, renal cell carcinoma (RCC), endometrial carcinoma, and hepatocellular carcinoma. Furthermore, co-blockade of TGF-β and PD-L1 was shown to reduce TGF-β signaling in stromal cells, facilitate T-cell infiltration into the tumor centers, provoking vigorous anti-tumor immunity and tumor regression [134]. Trials evaluating the combined inhibition of TGF-β and PD-(L)1 in a variety of solid tumors are underway (NCT04390763, NCT02423343). Compared to anti-PD-L1 alone, IL-10 inhibition improved responses to anti-PD-L1 and produced more cytotoxic effector KLRG1^+^, IFN-γ^+^, and memory CD8^+^ T-cells, and fewer exhausted T-cells in chronic lymphocytic leukemia [135]. In contrast, pegilodecakin (pegylated IL-10) has been tested in a clinical trial (NCT02009449) and demonstrated to have a manageable toxicity profile and preliminary anti-tumor activity when in combination with pembrolizumab or nivolumab in previously treated advanced RCC [136]. Hypoxia within the TME is an intrinsic property of all solid malignant tumors, and we have detailed how to target hypoxic TME synergistically with immunotherapy in another review [80]. The induction of tertiary lymphoid structures (TLSs) that represent local and favorable sites for generating antitumor humoral and cellular immune responses has been shown to be capable of overcoming resistance to ICIs [137, 138]. A number of such combination therapies are currently ongoing in clinical trials.

## Targeting epigenetic modification

Epi-drugs, defined as small-molecule inhibitors that target epigenetic regulators like DNMT, HDAC, and EZH2, have been shown to augment anti-tumor immune responses through multiple immunomodulatory activities [139, 140]. In animal models of breast, colon, and prostate cancers, inhibition of DNMT led to MHC-I upregulation, T-cell chemotaxis, and tumor infiltration of CD8^+^ T cells, thus enhancing the anti-tumor effects of anti-PD-1 antibodies [141]. Decitabine (a related DNMT inhibitor) stimulated the cytotoxic and infiltration responses of CD8^+^ T cells, which combined with anti-CTLA-4 antibody exhibited synergistic anti-tumor effects and extended survival in a syngeneic murine ovarian cancer model [142]. Panobinostat, an HDAC inhibitor, could upregulate the expression of PD-L1 and PD-L2 and improve anti-PD-1 immunotherapy in melanoma, resulting in improved inhibition of tumor growth and increased survival compared to anti-PD1 monotherapy [143]. Romidepsin was demonstrated to induce strong anti-tumor responses in a T cell-dependent manner and combinatory therapy with anti-PD-1 and induce tumor rejection in multiple lung tumor models [144]. Several preclinical models suggested that a novel orally bioavailable HDAC inhibitor HBI-8000 could augment the activity of ICIs targeting either PD-1, PD-L1, or CTLA-4, and significantly reduce tumor progression [145]. It has been shown that inhibiting EZH2 reversed acquired resistance and enhanced anti-tumor effects when in combination with anti-PD1, anti-CTLA-4, and IL-2 immunotherapy in prostate cancer and melanoma [146, 147]. Inspired by the encouraging results of preclinical studies, the combination therapy of ICIs with epigenetic drugs is being translated into clinical trials. A phase II trial (NCT02397720) revealed that the combination of azacitidine and nivolumab was safe and produced encouraging objective response rate and OS outcomes in patients with acute myeloid leukemia [148]. In phase I/Ib trial (NCT02638090), HDAC inhibitor vorinostat plus pembrolizumab was well tolerated and demonstrated preliminary anti-tumor activity, with three confirmed partial response (PR) and eleven stable disease responses among ICI-resistant metastatic NSCLC 24 patients [149]. Furthermore, preliminary results from the ENCORE-601 phase Ib/II trial revealed that entinostat, an experimental HDAC inhibitor, was well tolerated and, together with pembrolizumab, induced durable responses in melanoma patients whose disease progressed after PD-1 blockade monotherapy [150]. However, entinostat plus pembrolizumab provided a clinically meaningful benefit despite not achieving the primary response rate endpoint in the expansion cohort of ENCORE 601 that included anti-PD-(L)1-experienced patients with NSCLC [151]. Additionally, trials of EZH2 inhibitors plus pembrolizumab or ipilimumab are recruiting the patients with advanced solid tumors (NCT03854474 and NCT03525795). Together, these results provided a solid rationale for combining epigenetic agents with ICI immunotherapy.

## Targeting gut microbiome dysbiosis

Manipulating the gut microbiota has been shown to enhance anti-tumor immunity and alleviate the development of acquired resistance to ICIs [94, 152]. Sivan et.al first revealed that commensal *Bifidobacterium* can enhance anti-tumor immunity in vivo in a non-antigen-dependent manner, which exhibited synergistic anti-tumor effectiveness with PD-L1 inhibitor [153]. It was reported that oral feeding of *Bacteroides fragilis* with *Burkholderia cepacian* or *Bacteroides thetaiotaomicron* enhanced the efficacy of CTLA-4 blockade via promoting DC maturation and triggering Th1 immune responses [94]. Three bacterial species (*Lactobacillus johnsonii*, *Bifidobacterium pseudolongum*, and *Olsenella)* were found to significantly enhanced efficacy and promote ICI therapies by raising the activation of CD4^+^ and CD8^+^ T cells [154]. A study conducted in a mouse model suggested that the presence of *A. muciniphila* and *Akkermansia muciniphila* contributed to the immunogenicity of the PD-1 blockade, and its abundance was related to clinical responses. Fecal microbiota transplantations (FMT) restored the efficacy of anti-PD-1 blockade in an IL-12-dependent manner [89]. Motivated by the encouraging results of preclinical studies, numerous clinical trials focusing on how to modulate gut microbiota composition to overcome ICI resistance are ongoing. A phase I/II clinical trial (NCT03775850) revealed that EDP1503 (an oral microbiota product) combined with pembrolizumab was well-tolerated and safe, and biomarker analysis found that EDP1503 works though upregulating the CD8^+^ T cells/Treg cell ratio [155]. Results of the phase I/II trial (NCT03637803) investigating the synergistic efficacy of the oral probiotic MRx0518 combined with pembrolizumab in bladder cancer, NSCLC, RCC, or melanoma have not yet been published [156]. Results from two recent clinical trials (NCT03353402 and NCT03341143) have suggested that patients with metastatic melanoma-resistant to PD-1 inhibitor after the intervention of FMT from responders had improved clinical benefits [157, 158].

## Other emerging combination strategies

Besides discussed above, multiple emerging strategies combined with ICIs are underway, some of which reported early encouraging results. The stimulator of an interferon gene agonist-loaded lipid nanoparticles (STING-LNP) might represent a promising candidate for anti-PD-1-resistant tumors, which in combination with PD-1 blockade synergistically promoted anti-tumor response via the activation of NK cells to overcome anti-PD-1 resistance in a B16-F10 lung metastasis model [159]. Similarly, Li et al. reported that novel Microbubble-assisted UltraSound-guided Immunotherapy of Cancer (MUSIC) platform utilized antibody guided targeting to stimulating STING pathway in APCs, further sensitizing poorly immunogenic tumors to anti-PD-1 therapy [160]. Engineered nanoparticles carrying immunostimulatory oligonucleotides has been confirmed to synergize with anti-CTLA-4 therapy to achieve tumor suppression in animal models of melanoma, colon cancer, and breast cancer [161]. The tumor-targeted radioligand ^177^Lu-PSMA-617 has recently demonstrated anti-tumor activity in prostate cancer [162], and its synergy with anti–PD-1 is currently being investigated in a phase II trial (NCT03805594). In addition, the SARS-CoV-2 pandemic has enormously unlocked the potential of vaccine technology as a powerful therapeutic platform. Phase I trail (NCT02410733) suggested that BNT111 (an intravenously administered liposomal RNA vaccine), alone or in combination with PD1 blockade, mediated durable objective responses in ICI-experienced patients with unresectable melanoma [163]. Moreover, an exciting preclinical study demonstrated the vaccination of 8p4 + FK-33 nanoparticles (8FNs) incorporating different tumor antigens could inhibit tumor growth by reversing the immunosuppressive TME and potentiating anti-tumor immunity, exerting synergistic therapeutic effects with PD-1 inhibitor [164]. We hoped that the development of more emerging strategies could pave the way for overcoming resistance to ICI therapy.

## Future perspectives and conclusions

Although ICI therapy has revolutionized medical oncology, the emergence of acquired resistance in a considerable proportion of patients poses a significant challenge. Currently, the true landscape of acquired resistance to ICIs remains largely uncertain, thus the uniform definition and evaluation criteria are required to be established for acquired resistance. Moreover, emerging multifaceted approaches and higher resolution investigations of interactions involving host, tumor, and/or the TME may aid in the discovery new resistant mechanisms and predict new synergistic combinations. As ongoing research into underlying biology of acquired resistance to ICI therapy, the near future will witness more and more therapeutic combinations to overcome acquired resistance, resulting in clinical benefits for more patients. However, given the heterogeneity of tumor types and the complexity of anti-tumor immune responses, a wide set of biomarkers, such as TILs status, TMB, TLS, and PD-L1 expression, is required to predict the treatment efficacy or resistance to ICIs. With the development of human bioinformatic analysis and genome sequencing, more and more patients will definitely benefit from both integrated and individualized immunotherapies.

## Data Availability

Data sharing is not applicable to this article as no datasets were generated or analyzed during the current study.

## Abbreviations

ICIs: Immune checkpoint inhibitors
CTLA-4: Cytotoxic T lymphocyte antigen 4
PD-1: Programmed death 1
PD-L1: PD-ligand 1
OS: Overall survival
NSCLC: Non-small cell lung cancer
SITC: The Society for Immunotherapy of Cancer
APCs: Antigen-presenting cells
MHC-I: Major histocompatibility complex class I
TCR: T cell receptor
FDA: Food and Drug Administration
LAG3: Lymphocyte-activation gene 3
TILs: Tumor-infiltrating cells
ACT: Adoptive T-cell therapy
MANAs: Mutation-associated neoantigens
IFN-γ: Interferon-γ
EMT: Epithelial-to-mesenchymal transition
TGF-β: Transforming growth factor-β
IL-6: Interleukin-6
β2M: β-2-Microglobulin
JAK1/JAK2: Janus kinases 1 and 2
STAT: Signal transducer and activator of transcription
TIM3: T-cell immunoglobulin and mucin domain 3
BTLA: B and T lymphocyte attenuator
VISTA: V-domain Ig suppressor of T cell activation
TIGIT: T cell Ig and ITIM domain
MDSCs: Marrow-derived suppressor cells
Tregs: Regulatory T cells
TAMs: Tumor-associated macrophages;
CAFs: Cancer-associated fibroblasts
BTLA: B and T lymphocyte attenuator
TEFF: Effector T cells
CXCL12: Chemokine C-X-C motif ligand 12
VEGF: Vascular endothelial growth factor
DC: Dendritic cell
IDO: Indole 2,3-dioxygenase
PFS: Progression-free survival
EZH2: Zeste homolog 2
DNMT: DNA methyltransferase
TEX: Exhausted CD8+ T cells
TMEM: Memory T cells
irAEs: Immune-related adverse events
RCC: Renal cell carcinoma;
T-VEC: Talimogene laherparepvec
cIAP1/2: Cellular inhibitor of apoptosis proteins 1 and 2
NLRC5: Nucleotide-binding oligomerization domain-like receptor family caspase recruitment domain containing 5
CCR4: CC chemokine receptor 4
ATRA: All-trans-retinoic acid
CK2: Casein kinase 2
HCC: Hepatocellular carcinoma
TLSs: Tertiary lymphoid structures
SCL: Small cell lung cancer
HNSCC: Head and neck squamous cell carcinoma
cHL: Classical hodgkin lymphoma
PMBCL: Primary mediastinal large B cell lymphoma
MCC: Merkel cell carcinoma
TMB: Tumor mutational burden
CSCC: Cutaneous squamous cell carcinoma
TNBC: Triple-negative breast cancer
PDAC: Pancreatic ductal adenocarcinoma
STING-LNP: The stimulator of an interferon gene agonist-loaded lipid nanoparticles
MUSIC: Microbubble-assisted UltraSound-guided Immunotherapy of Cancer
iTPNCs: Immunostimulatory tandem peptide nanocomplexes
8FNs: 8P4 + FK-33 nanoparticles
